# Vitamin D status, hypertension and body mass index in an urban black community in Mangaung, South Africa

**DOI:** 10.4102/phcfm.v8i1.1210

**Published:** 2016-10-31

**Authors:** Ronette Lategan, Violet L. van den Berg, Jasminka Z. Ilich, Corinna M. Walsh

**Affiliations:** 1 Department of Nutrition and Dietetics, University of the Free State, South Africa; 2 Department of Nutrition, Food and Exercise Sciences, Florida State University, United States

## Abstract

**Background:**

A strong relationship exists between hypertension and body weight. Research has linked both higher blood pressure and body weight with lower vitamin D status.

**Objective:**

This study assessed the vitamin D status of a low-income, urban, black community in South Africa, to examine whether serum levels of 25-hydroxy vitamin D [25(OH)D] are associated with hypertension and body mass index (BMI).

**Methods:**

Data collected from 339 adults (25–64 years) from the Assuring Health for All in the Free State (AHA-FS) study were analysed. Variables measured include serum 25(OH)D, blood pressure, weight and height to determine BMI, and HIV status.

**Results:**

Mean 25(OH)D level was 38.4 ± 11.2 ng/mL for the group; 43.5 ± 11.8 ng/mL and 37.0 ± 10.6 ng/mL for males and females, respectively. Approximately 40% of the participants were HIV-positive and 63.4% hypertensive. Based on BMI, 11.8% were underweight, 33.0% normal weight, 23.0% overweight and 32.1% obese. HIV status showed no correlation with 25(OH)D levels when controlling for BMI. Poor inverse relationships were found between BMI and 25(OH)D (*p* = 0.01), and between mean arterial blood pressure and 25(OH)D (*p* = 0.05). When controlling for BMI, no correlation was found between 25(OH)D and the prevalence of hypertension or mean arterial blood pressure.

**Conclusion:**

Approximately 96% of participants had an adequate vitamin D status, which could be attributed to latitude, sunny conditions and expected high levels of sun exposure because of living conditions. Results confirmed a poor inverse relationship between vitamin D status and hypertension, which seems to be dependent on BMI.

## Introduction

Hypertension and obesity are global health concerns that affect morbidity and mortality in many communities. A strong relationship exists between body weight and the prevalence of hypertension. The risk of developing hypertension is two to six times higher in overweight than normal weight individuals, and evidence from almost all clinical trials recommends weight loss as first-line treatment for lowering blood pressure.^1,2,3^ In recent years, vitamin D has become a nutrient of interest, with researchers investigating its role in various disease conditions. Although vitamin D status and its role in many non-skeletal health conditions have been investigated widely, its relationship with chronic conditions, including hypertension and obesity, still remains controversial,^4^ with only a few studies performed in black South Africans.

An inverse relationship, often age related, between vitamin D status and systolic blood pressure has been described and links lower serum 25-hydroxy vitamin D [25(OH)D] levels with a higher risk of hypertension.^5,6,7,8^ Vitamin D seems to act as a negative regulator of the renin gene,^9^ with a vitamin D deficiency promoting an increase in blood pressure by increasing the expression of renin. Renin activates angiotensin II, which increases blood pressure by direct vasoconstriction and by causing sodium and water retention in the kidney.^8,9^ A meta-analysis by the Endocrine Society Task Force, however, concluded that evidence of vitamin D improving cardiovascular risk factors is weak or non-existent.^10^ A review by Wang^11^ concluded that maintenance of an adequate vitamin D status is beneficial to cardiovascular and cerebrovascular health. However, as Vaidya and Forman^5^ cautioned in their review, supplementation with vitamin D in many studies has failed to lower blood pressure levels, and the relationship should be investigated further before vitamin D is recommended as a treatment option.

An inverse relationship between body mass index (BMI) and vitamin D status has been described.^12,13,14^ Wortsman et al.^15^ have shown that both obese and normal weight persons’ skin produces the same amount of vitamin D under the same conditions, but that 57% less vitamin D is absorbed into the circulation of obese persons, because of the higher amount of subcutaneous fat that traps the cholecalciferol. Sun exposure in obese individuals with a low vitamin D status, therefore, does not seem to be the solution for normalising vitamin D status, and it should rather be supplemented orally so that it is released into circulation before being stored in the adipose tissue.^15^

Although HIV infection may influence body weight, which in turn may have an effect on vitamin D levels, similar mean serum 25(OH)D levels have been reported in HIV-infected and non-infected individuals.^16^

The amount of time that skin needs to be exposed to sun in order to obtain adequate ultraviolet B (UVB) exposure for sufficient production of vitamin D3 in human skin depends on the solar elevation angle (time of day and latitude), as well as surface and atmospheric conditions.^17^ For a darker pigmented skin, the need for sun exposure to produce adequate vitamin D can be up to 40 times longer than what is needed by a fair skin because of the higher amount of cutaneous melanin in darker pigmented skin, which slows conversion to cholecalciferol (pre-vitamin D) in the skin. For a darker pigmented skin, estimated exposure times in central South Africa (latitude 29°10´S for Mangaung) vary between 136 min at 09:00 in the winter to 16 min at 12:00 in the summer, based on a quarter of body surface exposed (arms, hands and face).^13,17^

This article reports on the vitamin D status of a low-income, black, urban community in Mangaung, South Africa, and discusses the relation between vitamin D status and the prevalence of hypertension and BMI, taking into account possible confounding factors, such as HIV status. We hypothesised that 25(OH)D levels in this population will be inadequate because of low dietary intake and dark skin of participants, and that vitamin D status will be inversely correlated with blood pressure and BMI.

## Methods

For this cross-sectional study, baseline data from the Assuring Health for All in the Free State (AHA-FS) study in an urban setting were used. Data were collected in autumn, over a period of 2 weeks from 6 urban township areas in Bloemfontein, South Africa. Households were selected by proportional cluster sampling, stratified by area and formal plot/squatter households in open areas. Using randomly selected X and Y coordinates, 100 starting points were selected. From each starting point, five adjacent households were invited to participate and written informed consent obtained from all eligible adults (25–64 years). Participants assembled at the central research centre after an overnight fast, where blood pressure and anthropometric measurements were obtained, and blood samples drawn to assess vitamin D and HIV status.

Blood pressure was measured by a registered medical practitioner according to recognised guidelines.^18^ Hypertension was defined as systolic blood pressure of ≥ 140 mmHg and/or diastolic blood pressure ≥ 90 mmHg.^2^ Participants using prescription medication for the management of hypertension at the time of the consultation were also classified as hypertensive.^19^ The mean arterial pressure was calculated as follows: diastolic blood pressure + ⅓(systolic blood pressure – diastolic blood pressure).^20^

Anthropometric measurements were taken by trained final-year dietetics students, under supervision of the researchers. Body weight was determined using WHO guidelines^21^ on a floor-type Seca 770 digital scale (Medical Scales and Measuring Systems, Seca kk., Japan) with accuracy to 100 g and a maximum capacity of 200 kg. Height was measured with a Seca stadiometer (Medical Scales and Measuring Systems, Seca kk., Japan) accurate to the nearest 5 mm.^21^ BMI (kg/m^2^) was calculated and interpreted according to WHO guidelines.^22^

HIV status was determined from fasting venous blood samples after counselling and informed consent was obtained. Primary screening for HIV was performed using the Enzygnost HIV Integral II Ag/Ab test (Dade Behring, Marburg, Germany). Results were confirmed by the Vironostika HIV Uni-Form II Ag/Ab test (bioMérieux, Marcy l’Etoile, France) and participants could indicate whether they wished to know their status. Incidence reported for HIV infection in South Africa varies and is estimated at more than 17%.^23,24^ Although HIV infection influences body weight, which is associated with vitamin D status (particularly in obese individuals), similar mean serum 25(OH)D levels have been reported in HIV-positive and -negative individuals.^16^

A chemiluminescent immunoassay, as determined by means of the Liaison 25(OH) Vitamin D Total Assay Kit (DiaSorin, Stillwater, MN, USA), was used to measure serum 25(OH)D levels. Although a serum level of lower than 30 ng/mL 25(OH)D was previously used to indicate a deficiency,^25^ the Health and Medicine Division of the National Academies of sciences, Engineering and Medicine recently lowered the cut-off value for adequate vitamin D status to 20 ng/mL.^26^ For the purpose of this study, a serum 25(OH) D level of more than 20 ng/mL indicated an acceptable vitamin D status, while a value of 12–20 ng/mL indicated inadequacy, and a level lower than 12 ng/mL indicated deficiency.^26^

Analysis of data was performed with PASW (Predictive Analytics SoftWare) statistics software by SPSS (Version 18.0). Frequencies and percentages were used to express categorical data. Means and standard deviations, or percentiles, as appropriate, were used to express quantitative variables. Comparisons of means were performed using *t*-tests. Chi-square tests, two-tailed Pearson correlations and multivariate logistic regression models were used to describe and test associations between variables. A *p* ≤ 0.05 was considered statistically significant.

## Ethical considerations

Ethical approval was obtained from the Ethics Committee of the Faculty of Health Sciences, University of the Free State (ETOVS: 21/07).

## Results

In this study, 339 adults (76 males and 263 females) with complete data sets were included, with a mean age of 44.3 years, ranging between 25 and 64 years (Table 1). More than a third (39.8%) of the sample was HIV-positive. In 41.6% of individuals, systolic blood pressure was ≥ 140 mmHg, while 46.6% had a diastolic blood pressure of ≥ 90 mmHg, indicating hypertension. Approximately one quarter (25.4%) of participants used antihypertensive medication at the time of the study. Consequently, 63.4% (57.9% of male and 65.0% of female participants) were classified as hypertensive.

**TABLE 1:** General description of the study population in terms of age, blood pressure, body mass index (BMI) and 25-hydroxy vitamin D [25(OH)D] levels.

Variable	*N*	Mean ± s.d.	Minimum	Maximum
Anthropometric measurements				
Age (years)	339	44.3 ± 10.6	25.0	63.0
Height (cm)	339	159.4 ± 7.8	139.8	180.0
Weight (kg)	339	70.1 ± 21.4	31.9	140.0
Blood pressure				
Systolic (mmHg)	339	135.5 ± 23.7	72.0	203.0
Diastolic (mmHg)	339	89.8 ± 17.6	46	188
BMI (kg/m^2^)				
Male	76	21.4 ± 5.6	14.5	49.9
Female	263	29.6 ± 8.7	13.3	55.7
Total	339	27.8 ± 8.8	13.3	55.7
25(OH)D level (ng/mL)				
Male	76	43.5 ± 11.8	15.6	82.2
Female	263	37.0 ± 10.6	8.7	64.8
Total	339	38.4 ± 11.2	8.7	82.2

*Source:* Authors’ own work

s.d., standard deviation.

The mean 25(OH)D level for the group was 38.4 ng/mL and was significantly higher in males than in females (43.5 and 37.0 ng/mL, respectively; *p* < 0.001).

A high incidence of overweight/obesity was found in this study population. Based on BMI, 11.8% of the study population was underweight (25.0% of males and 8.0% of females), 33.0% had a normal BMI, 23.0% was overweight and 32.1% obese. More than half (55.1%) of the participants were either overweight or obese according to their BMI, with the majority of males (81.6%) classified as underweight or normal weight and the majority of females (65.8%) classified as overweight or obese.

**FIGURE 1:**
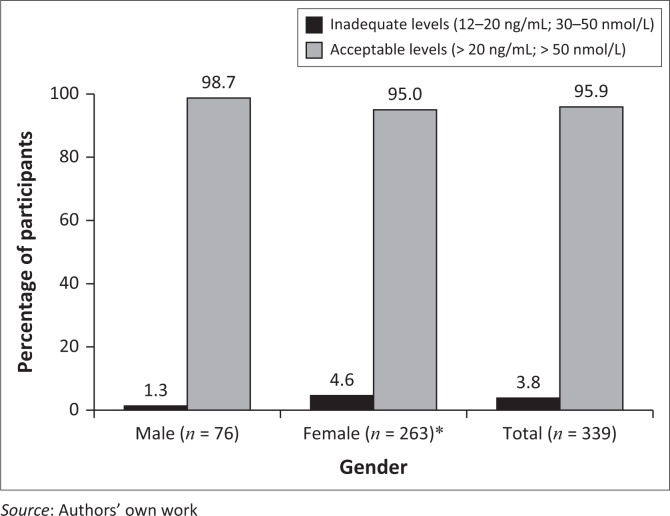
Vitamin D status of participants.

The majority of the participants in this study had an acceptable vitamin D status, as indicated in Figure 1. Only one (0.4%) female participant, which constituted 0.3% of the total population, had a deficient level of 25(OH)D (< 12 ng/mL; < 30 nmol/L). Approximately 96% of the total group of participants had 25(OH)D levels of > 20 ng/mL (> 50 nmol/L).

**FIGURE 2:**
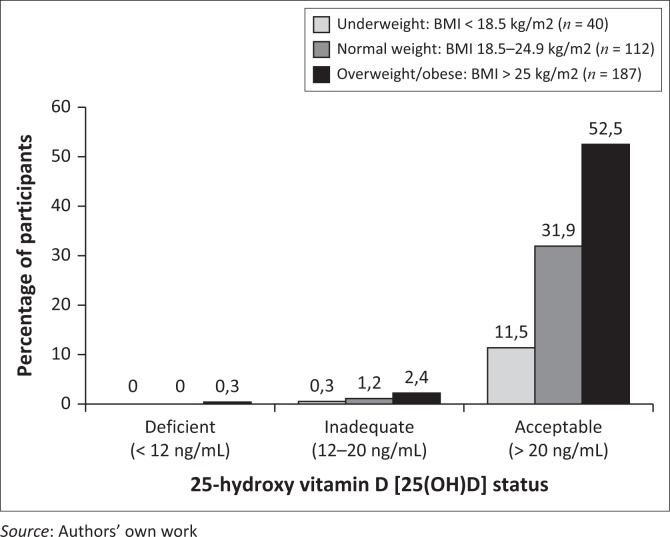
Vitamin D status in relation to body mass index (BMI) category.

In Figure 2, participants’ vitamin D status, classified as deficient, inadequate and acceptable, is indicated according to BMI category, showing a tendency of an increase in the prevalence of lower vitamin D status as BMI category increases. A Pearson’s correlation showed an inverse relationship (*r* = −0.303; *p* < 0.001) between BMI and 25(OH) D levels as a measure of vitamin D status.

The large majority of participants (95.9%) had an acceptable vitamin D status. No association between vitamin D status and the prevalence of hypertension was found. Of the hypertensive individuals, 95.8% had an acceptable vitamin D status, while 96.0% of the normotensive individuals had an acceptable vitamin D status.

A Pearson’s correlation of mean arterial blood pressure and 25(OH)D levels showed a very weak inverse correlation (*r* = −0.136; *p* = 0.012). Given the influence of overweight/obesity, as indicated by BMI, on both 25(OH)D levels and blood pressure, the association between 25(OH)D levels and mean arterial blood pressure was determined, controlling for BMI. No significant correlation between 25(OH)D levels and hypertension (*r* = −0.021; *p* = 0.694) or mean arterial blood pressure levels (*r* = −0.062; *p* = 0.254) were found.

More than a third (39.8%) of the study population was HIV-positive, although HIV status did not correlate significantly with 25(OH)D (*r* = 0.101; *p* = 0.06) when BMI was accounted for.

## Discussion

In this study, a high prevalence of hypertension (63.4%) and overweight/obesity (55.1%) was found. Approximately 96% of participants had adequate vitamin D status (> 20 ng/mL), which was not expected, because of the darker skin pigmentation of participants^17^ and expected low vitamin D intake because of the lower socio-economic area^27^ and absence of compulsory vitamin D fortification in the country.

The high incidence of HIV infection did not correlate with 25(OH)D levels, except through the indirect influence on weight and BMI. This finding was in agreement with Stephensen et al.^16^ who found no significant difference between the mean serum 25(OH)D levels of HIV-positive and HIV-negative individuals.

An inverse relationship between 25(OH)D levels and both BMI and mean arterial blood pressure was found. However, when controlled for BMI, no significant relationship was found between 25(OH)D and hypertension or mean arterial blood pressure, indicating that body weight may be a confounding factor in the relationship between vitamin D levels and hypertension.

When comparing this population, from a low socio-economic, disadvantaged background,^27^ with populations from developed countries where foods are also commonly fortified with vitamin D, it was surprising that the mean serum 25(OH)D level of 38.4 ng/mL in this population was significantly higher (*p* < 0.001) than that reported from the US National Health and Nutrition Examination Survey (males 25.2 ng/mL; females 24.6 ng/mL; black population 15.7 ng/mL),^13^ or that reported for participants from the Prostate Lung, Colorectal, and Ovarian Cancer Screening Trial cohort (males 24.4 ng/mL; females 26.2 ng/mL).^12^ As in other studies, higher mean 25(OH)D levels were found in males compared to females (43.5 ng/mL versus 37.0 ng/mL).^13,28^

Similar to the high proportion of individuals with adequate vitamin D status found in this adult population, Poopedi et al.^29^ also reported that 74% of a population of South African children aged 10 years old, living in the urban area of Johannesburg, had adequate vitamin D status (using cut-off values of ≥ 75 nmol/L or ≥ 30 ng/mL). The mean serum 25(OH)D levels for the black children in the study were 34.5 ng/mL for girls and 40.1 ng/mL for boys,^29^ which agrees with the higher levels found in the black adults in the current study.

In our study, a notably higher vitamin D status, compared to studies from other countries, was found. A much lower vitamin D status was expected because of the dark skin colour of the study population, low income levels^27^ translating to low intakes of vitamin D-rich foods, the fact that vitamin D fortification is not mandatory in South Africa, and the large incidence of overweight/obesity in this study group. The high levels of 25(OH)D may probably be ascribed to the favourable latitude (29°10´S) of and general sunny weather experienced in Bloemfontein, South Africa; the fact that blood samples were taken during autumn, and the fact that living conditions and lifestyles of this community allow high levels of sun exposure, linked to outdoor living conditions.
